# P-1533. Assessing the Impact of Antimicrobial Stewardship on Antibiotic De-escalation, Escalation, Discontinuation, and Continuation: A Prospective Observational Study

**DOI:** 10.1093/ofid/ofae631.1701

**Published:** 2025-01-29

**Authors:** Suresh Kumar Dorairajan, R Sanjai, S Shibi, S Shobana

**Affiliations:** Apollo hospital, Chennai, Tamil Nadu, India; Apollo Hospital, Chennai, Tamil Nadu, India; Apollo Hospital, Chennai, Tamil Nadu, India; Apollo Hospital, Chennai, Tamil Nadu, India

## Abstract

**Background:**

This study focuses on the involvement of infectious disease (ID) specialists in patient care regarding the de-escalation, escalation, discontinuation, and continuation of antibiotics, aimed at reducing unnecessary antibiotic usage, in alignment with the World Health Organization (WHO) AWaRe classification

**Methods:**

This prospective observational study was conducted over the month of March 2024 at a 240-bed tertiary hospital in South India. Infectious disease (ID) consultants were actively involved in patient assessment to evaluate the appropriateness of antibiotic prescriptions. Following their assessment, ID consultants made decisions regarding antibiotic management, including de-escalation, escalation, discontinuation, and continuation, based on the patient's clinical condition. Data on antibiotic usage and outcomes, including appropriateness and clinical response, were collected using a simple audit tool. These data were subsequently entered into an Excel spreadsheet and analysed using descriptive statistics.

**Results:**

Out of [1141] patients admitted to the tertiary care hospital, [597] patients received antibiotics, in that [80] were seen by infectious disease (ID) consultants. Among these, [30] patients underwent de-escalation, [10] experienced escalation, [28] had discontinuation, and [12] underwent continuation of antibiotic therapy

**Table:** 

Category	Frequency	percentage
Total patients admitted	1141	100%
Patients received antibiotics	597	52%
Infectious disease consultant seen	80	7%
De- escalation	30	3%
Escalation	10	0.87%
Discontinuation	28	3%
Continuation	12	1%

**Conclusion:**

The study highlights the critical role of infectious disease specialists in guiding antibiotic therapy decisions, which can lead to more personalized and effective patient care. By reducing unnecessary antibiotic usage and promoting antimicrobial stewardship, this research contributes to improving patient outcomes and combating antimicrobial resistance.

**Disclosures:**

**All Authors**: No reported disclosures

